# A new species of *Acetalius* Sharp from eastern China (Coleoptera, Staphylinidae, Pselaphinae)

**DOI:** 10.3897/zookeys.592.8769

**Published:** 2016-05-25

**Authors:** Zi-Wei Yin, Li-Zhen Li

**Affiliations:** 1 Department of Biology, Shanghai Normal University, 100 Guilin Road, Shanghai, 200234, P. R. China

**Keywords:** Acetalius, new species, eastern China, Asia, key, taxonomy

## Abstract

The genus *Acetalius* Sharp currently contains two species from Japan. In this paper, a third species, *Acetalius grandis* Yin & Li, **sp. n.**, is described from eastern China. The foveal pattern of *Acetalius*, and polymorphism and major diagnostic features of *Acetalius grandis* are figured. An updated key to *Acetalius* species is provided.

## Introduction

The genus *Acetalius* Sharp was originally described from a single species, *Acetalius dubius* Sharp, based on a male collected from leaf litter in Kyushu (Suwa Shrine), Japan (Sharp 1883). The specific epithet (*dubius* means ‘doubtful, dubious’) reflected the author’s uncertainty about the higher placement of this genus, by stating that the new species has a ‘Pselaphini-like hind body’ and an ‘elongate *Euplectus* head’. Since the original description, *Acetalius* has been historically placed in the tribe Brachyglutini (Raffray 1903, 1904a, 1904b, 1904c 1908, 1911), in its own tribe Acetaliini (Jeannel 1958), or together with *Philoscotus* Sawada in subtribe Acetaliina of Euplectini (Besuchet 1985, Nomura 1988a, Newton and Chandler 1989). Recently, Chandler (2001) restricted the concept of Euplectini, removed all taxa having visible tergites IX and sternite IX to the Trichonychini, and placed Acetaliina as a junior synonym of Panaphantina.

Besuchet (1985) redescribed *Acetalius dubius* and added a second species, *Acetalius pilosus* Besuchet, based on a single male from Shikoku. Thereafter, both species of *Acetalius* were treated by Nomura in a series of papers (Nomura 1988a, b, c) revising the subtribe Acetaliina. Thus *Acetalius* is so far represented by two species confined to Japan (Nomura 2013). Members of *Acetalius* lack distinct abdominal paratergites, which are reduced to pairs of marginal carinae, and lack the lateral metacoxal foveae that are often present in other genera of Panaphantina. During our investigation of the pselaphine fauna of eastern China, a third species of *Acetalius* was recognized among the material collected from Fengyangshan Nature Reserve, Zhejiang, which is described in this paper. The entire foveal pattern of *Acetalius* is investigated and described on the basis of a disarticulated male paratype on a slide preparation.

## Material and methods

The type material treated in the present paper is deposited in the Insect Collection of the Shanghai Normal University (SNUC).

Dissected parts were preserved in Euparal mounting medium on a plastic slide that was placed on the same pin with the specimen. To investigate the foveal pattern, a male paratype was completely disarticulated and preserved on a slide preparation. Habitus images were taken using a Canon 5D Mark III camera in conjunction with a Canon MP-E 65mm f/2.8 1-5X Macro Lens and a Canon MT-24EX Macro Twin Lite Flash. Images of the morphological details were made using a Canon G9 camera mounted on an Olympus CX31 microscope. Line drawings were initially produced using an Olympus U-DA Drawing Tube, and then inked in Adobe Illustrator CS5. Zerene Stacker version 1.04 was used for image stacking. All images were modified and grouped in Adobe Photoshop CS5 Extended.

The collecting data of the material are quoted verbatim, information not included on the label is placed in parentheses. A slash is used to separate different labels.

The following abbreviations are applied: AL – length of the dorsally visible part of the abdomen (posterior to elytra) along the midline; AW – maximum width of the abdomen; EL – length of the elytra along the suture; EW – maximum width of the elytra; HL – length of the head from the anterior clypeal margin to the occipital constriction; HW – width of the head across the eyes; PL – length of the pronotum along the midline; PW – maximum width of the pronotum. Length of the body (BL) is a combination of HL + PL + EL + AL.

## Systematics

### Acetalius grandis

Taxon classificationAnimaliaColeopteraStaphylinidae

Yin & Li sp. n.

http://zoobank.org/2C9DA199-AEE1-4601-9446-E6470AB18F98

#### Type material.

**Holotype: macropterous** ♂: ‘China: S. Zhejiang, Longquan (龙泉市), Fengyang Shan (凤阳山), creek valley nr. hotel, 27°54'42.85"N, 119°11'52"E, leaf litter, wood, sifted, 1190–1250 m, 28.iv.2014, Peng, Song, Yan, Yin, & Yu leg. / HOLOTYPE [red] ♂, *Acetalius grandis* sp. n., det. Z.-W. Yin, 2016’ (SNUC). **Paratypes: 1 apterous** ♂, **1 apterous** ♀, same collecting data as the holotype (SNUC); **1 apterous** ♂, **3 apterous** ♀♀: ‘China: S. Zhejiang, Longquan, Fengyang Shan, Da-Tian-Ping (大田坪), 27°54'36"N, 119°10'20"E, leaf litter, moss, ferns, sifted & beating, 1320 m, 27.iv.2014, Peng, Song, Yan, Yin, & Yu leg.’ (SNUC). Each paratype bears a yellow type label similar to holotype except for ‘PARATYPE, ♂ (or ♀)’.

#### Diagnosis.

Body large-sized, 1.85–2.23 mm; frons with a Y-shaped carina extending anteriorly to reach clypeal anterior margin; each eye of macropterous male with about 55 facets, that of apterous male with about 25–30 facets, and apterous female about 12 facets; antennomeres III elongate, IV–X distinctly transverse; abdominal tergite IV with three pairs of marginal carinae, discal carinae parallel; sternite IV with long median and two shorter admesal carinae.

#### Description.

**Figure 1.**
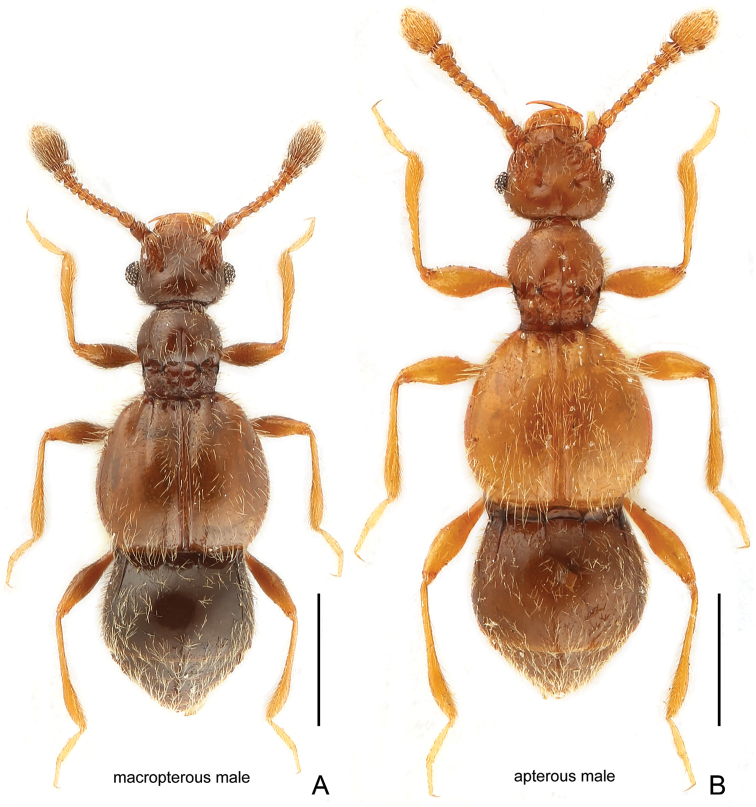
Dorsal habitus of *Acetalius grandis*. **A** Macropterous male **B** Apterous male. Scale bars: 0.5 mm.

**Figure 2.**
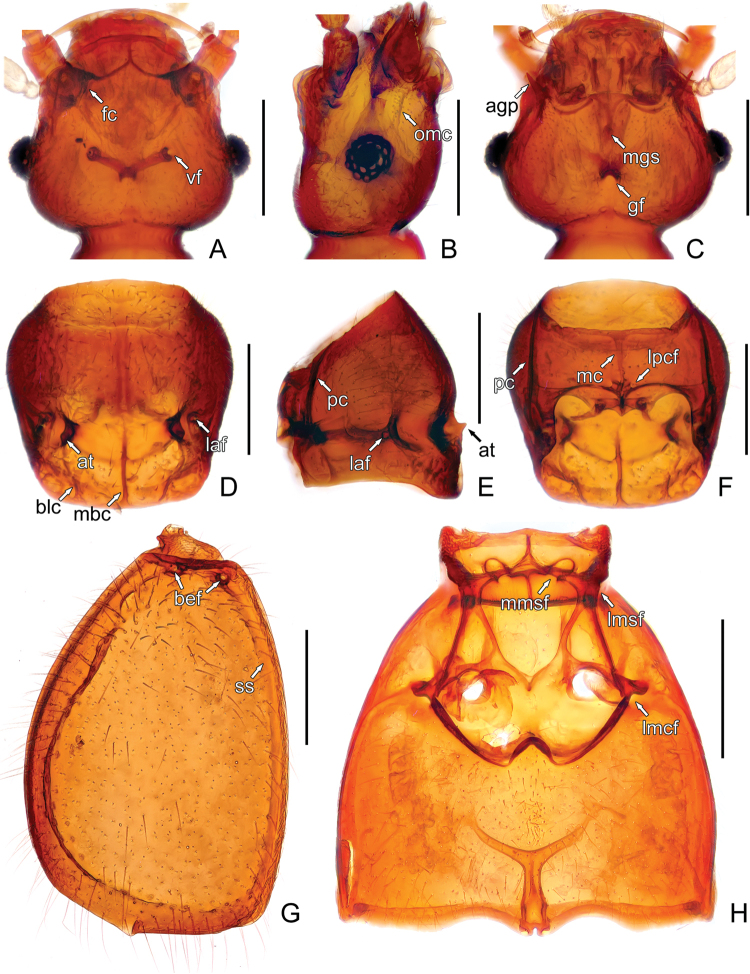
Morphology of *Acetalius grandis*. **A** Head, in dorsal view **B** Same, in lateral view **C** Same, in ventral view **D** Pronotum **E** Prothorax, in lateral view **F** Prosternite **G** Left elytron **H** Meso- and metaventrite. Abbreviations: agp – apicolateral genal projections, at – antebasal tubercles, bef – basal elytral foveae, blc – basolateral carinae, fc – frontal carina, gf – gular fovea, laf – lateral antebasal foveae, lmcf – lateral mesocoxal foveae, lmsf – lateral mesoventral foveae, lpcf – lateral procoxal foveae, mbc – mediobasal carina, mc – median carina, mgs – median gular sulcus, mmsf – median mesoventral foveae, omc – ocular-mandibular carinae, pc – paranotal carinae, ss – sutural striae, vf – vertexal foveae. Scale bars: 0.2 mm.

**Figure 3.**
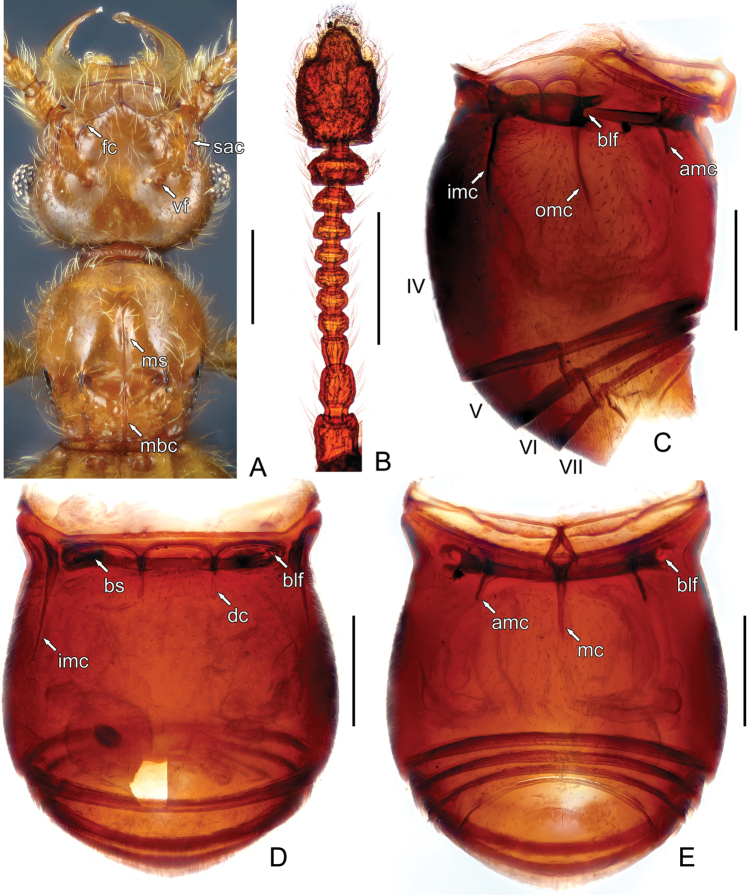
Morphology of *Acetalius grandis*. **A** Head dorsum and pronotum **B** Right antenna **C** Abdomen, in lateral view **D** Abdomen, in dorsal view **E** Abdomen, in ventral view. Abbreviations: amc – admesal carinae, blf – basolateral foveae, bs – basal carina, bs – basal sulcus, dc – discal carinae, fc – frontal carina, imc – inner marginal carinae, mbc – mediobasal carina, ms – median sulcus, omc – outer marginal carinae, sac – subantennal carinae, vf – vertexal foveae. Scale bars: 0.2 mm.

Apterous male (Fig. 1B). Length 1.91–2.03 mm; head (Fig. 2A–C) roundly rectangular, HL 0.34–0.36 mm, HW 0.43–0.46 mm; with small, nude vertexal foveae (Figs 2A, 3A; *vf*) situated at dorsal level of midline of eyes, lacking sulcus connecting vertexal foveae; antennal tubercles moderately prominent, connected by Y-shaped frontal carina (Figs 2A, 3A; *fc*) that extends anteriorly to meet anterior margin of clypeus, area between antennal tubercles depressed; with pair of subantennal carinae (Fig. 3A; *sac*) just posterior of antennal bases; each eye composed of about 25–30 facets; mandibles exceptionally elongate; antennal clubs (Fig. 3B) formed by apical two antennomeres, antennae with large scapes and pedicles, antennomeres III elongate, IV–X strongly transverse, successively wider and larger, XI largest, nearly oval, apex forming thumb-like protuberance surrounded by ring of thick setae; ocular-mandibular carinae (Fig. 2B; *omc*) present; gular foveae (Fig. 2C; *gf*) in single pit, situated in strongly depressed area, median gular sulcus (Fig. 2C; *mgs*) thin; apicolateral genal projections (Fig. 2C; *agp*) present.

Pronotum (Fig. 2D–F) as long as wide, PL 0.35–0.37 mm, PW 0.35–0.36 mm, laterally rounded at apical half, sides of basal half successively constricted toward base; broad antebasal sulcus connecting nude lateral antebasal foveae (Fig. 2D–E; *laf*); lacking median antebasal fovea; disc moderately convex, with antebasal tubercles (Fig. 2D–E; *at*) in sulcus, mediobasal carina (Figs 2D, 3A; *mbc*) extending from anterior margin of antebasal sulcus to pronotal base, with pair of short basolateral carinae (Fig. 2D; *blc*); median longitudinal sulcus (Fig. 3A; *ms*) thin, lacking lateral longitudinal sulci; paranotal carinae (Fig. 2E–F; *pc*) sinuate, complete. Prosternite with distinct median carina (Fig. 2F; *mc*); lateral procoxal foveae small and close (Fig. 2F; *lpcf*).

Elytra (Fig. 2G) rounded laterally, distinctly constricted at base, EL 0.62–0.64 mm, EW 0.66–0.71 mm; each elytron with two basal foveae (Fig. 2G; *bef*); with one pair of short discal striae extending from inner margins of outer basal foveae posteriorly to less than one-fourth of elytral length; sutural striae (Fig. 2G; *ss*) complete; lacking subhumeral foveae, marginal sulci, and apicolateral cleft. Metathoracic wings absent.

Mesoventrite with two widely separated median foveae (Fig. 2H; *mmsf*); lateral mesoventral foveae (Fig. 2H; *lmsf*) simple, slanted anteriorly; with lateral mesocoxal foveae (Fig. 2H; *lmcf*); metaventrite lacking lateral metacoxal foveae, posterior margin with narrow split at middle.

Legs with profemora simple ventrally, protibiae slightly expanded at apices.

**Figure 4.**
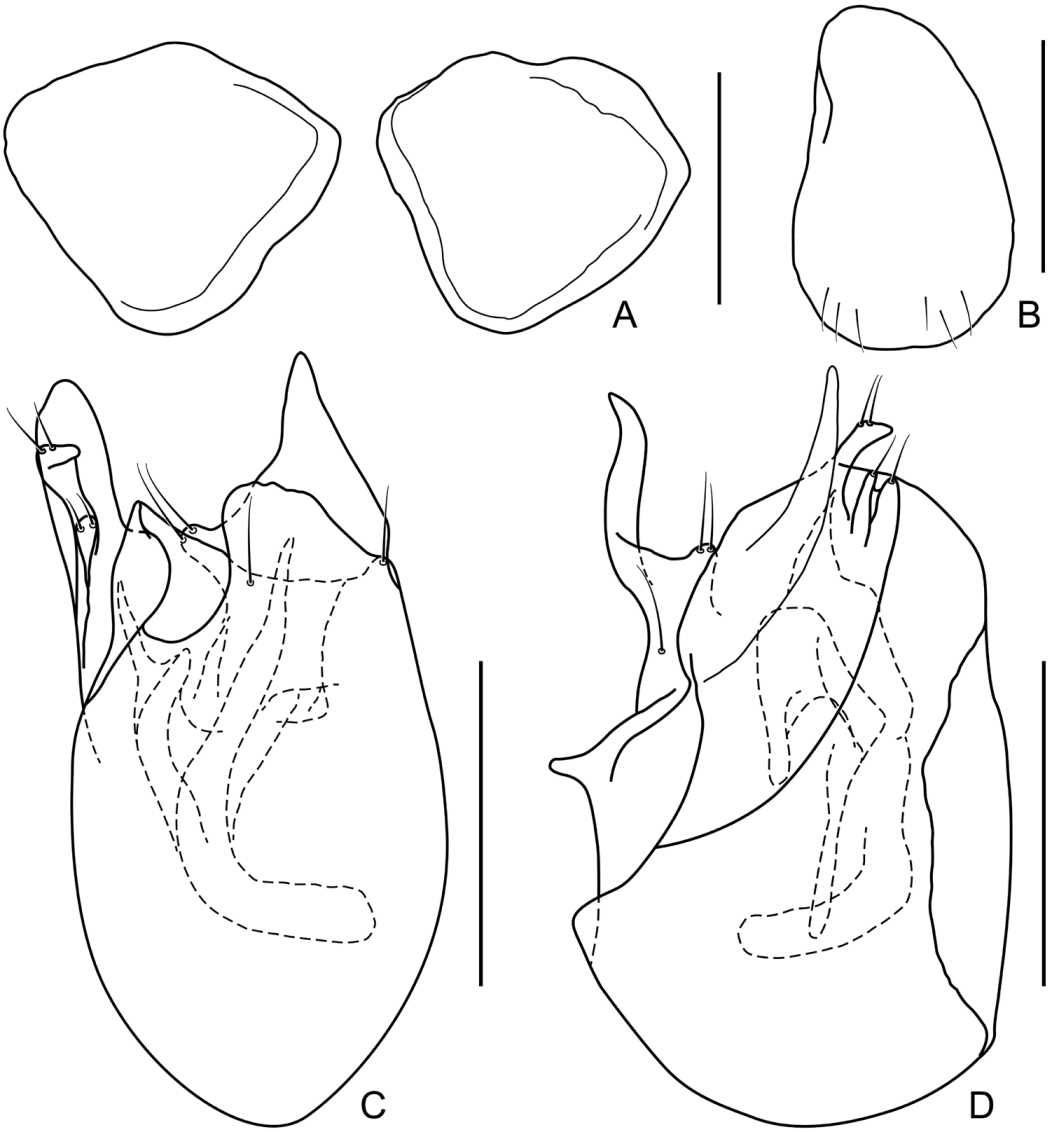
Morphology of *Acetalius grandis*. **A** Male tergites IX **B** Male sternite IX (penial plate) **C** Aedeagus, in dorsal view **D** Same, in lateral view. Scale bars: 0.1 mm.

Abdomen (Fig. 3C–E) widest at middle, AL 0.60–0.66 mm, AW 0.61–0.67 mm, with tergite IV (first visible tergite) longest, V–VIII successively shorter. Tergite IV with two small basolateral foveae (Fig. 3D; *blf*) in deep basal sulcus (Fig. 3D; *bs*) which is separated into three parts by pair of short, parallel discal carinae (Fig. 3D; *dc*), with short pair of short carinae between long inner and outer marginal carinae (Fig. 3C–D; *imc*, *omc*); V–VIII each with one pair of small basolateral foveae; tergites IX (Fig. 4A) paired, weakly sclerotized. Sternite III (first visible sternite) short, with arrow-like protuberance at middle; IV longest, with two large basolateral foveae (Fig. 3E; *blf*) in basal impression, with single long median carina (Fig. 3E; *mc*) and pair of shorter admesal carinae (Fig. 3E; *amc*); sternites V–III each short; sternite IX (Fig. 4B) nearly oval.

Length of aedeagus (Fig. 4C–D) 0.25–0.26 mm; aedeagus weakly sclerotized, parameres fused with median lobe; endophallus composed of one large, curved and several smaller sclerites.

Macropterous male (Fig. 1A). Measurements: BL 1.85 mm, HL 0.32 mm, HW 0.42 mm, PL 0.32 mm, PW 0.32 mm, EL 0.58 mm, EW 0.66 mm, AL 0.63 mm, AW 0.58 mm. Length of aedeagus 0.24 mm. Each eye composed of about 55 facets; base of elytra more expanded than that of apterous male, each elytron with two discal striae extending from lateral and mesal margin of outer basal foveae, respectively. Metathoracic wings fully developed.

Apterous female. Measurements: BL 2.19–2.23 mm, HL 0.40–0.42 mm, HW 0.48–0.50 mm, PL 0.39–0.42 mm, PW 0.36–0.38 mm, EL 0.66–0.72 mm, EW 0.76–0.80 mm, AL 0.70–0.71 mm, AW 0.70–0.75 mm. Each eye composed of about 12 facets. Elytral base constricted as that in apterous male. Metathoracic wings absent. Apices of protibiae not expanded.

#### Comparative notes.

*Acetalius grandis* can be readily separated from both *Acetalius dubius* (1.1–1.4 mm) and *Acetalius pilosus* (1.4–1.6 mm) by the distinctly larger body size (1.85–2.23 mm), and presence of a frontal carina on the head, which is lacking in the other species. The new species shares with *Acetalius pilosus* the presence of three pairs of marginal carinae on tergite IV, and similar structure of the aedeagus, but differs also by the elongate antennomeres III, which are as long as wide in *Acetalius pilosus*. *Acetalius dubius* has the smallest body size, and there are only two pairs of marginal carinae on tergite IV.

#### Distribution.

Eastern China: Zhejiang.

#### Etymology.

The specific epithet implies that *Acetalius grandis* is the largest species in the genus.

#### Key to *Acetalius* species

(modified from Nomura 1988a)

**Table:** 

1	Body size 1.85–2.23 mm; frons with a Y-shaped carina extending anteriorly to reach anterior margin of the clypeus. (Eastern China: Zhejiang)	***Acetalius grandis* Yin & Li, sp. n.**
–	Body size 1.1–1.6 mm; frons lacking carina	**2**
2	Body size relatively smaller, 1.1–1.4 mm; tergite IV with two pairs of marginal carinae, discal carinae slightly divergent; female has eyes each with 2–3 facets. (Japan: Honshû, Shikoku, Kyûshû, Tsushima)	***Acetalius dubius* Sharp**
–	Body size relatively larger, 1.4–1.6 mm; tergite IV with three pairs of marginal carinae, discal carinae strictly parallel; female has eyes each with about 20 facets. (Japan: Shikoku, Kyûshû)	***Acetalius pilosus* Besuchet**

## Supplementary Material

XML Treatment for Acetalius grandis
